# Building the microbiome in health and disease: niche construction and social conflict in bacteria

**DOI:** 10.1098/rstb.2014.0298

**Published:** 2015-08-19

**Authors:** Luke McNally, Sam P. Brown

**Affiliations:** 1 Centre for Immunity, Infection and Evolution, University of Edinburgh, Edinburgh EH9 3FL, UK; 2 Institute of Evolutionary Biology, School of Biological Sciences, University of Edinburgh, Edinburgh EH9 3FL, UK

**Keywords:** social evolution, niche construction, cooperation, microbiota, community ecology

## Abstract

Microbes collectively shape their environment in remarkable ways via the products of their metabolism. The diverse environmental impacts of macro-organisms have been collated and reviewed under the banner of ‘niche construction’. Here, we identify and review a series of broad and overlapping classes of bacterial niche construction, ranging from biofilm production to detoxification or release of toxins, enzymes, metabolites and viruses, and review their role in shaping microbiome composition, human health and disease. Some bacterial niche-constructing traits can be seen as extended phenotypes, where individuals actively tailor their environment to their benefit (and potentially to the benefit of others, generating social dilemmas). Other modifications can be viewed as non-adaptive by-products from a producer perspective, yet they may lead to remarkable within-host environmental changes. We illustrate how social evolution and niche construction perspectives offer complementary insights into the dynamics and consequences of these traits across distinct timescales. This review highlights that by understanding the coupled bacterial and biochemical dynamics in human health and disease we can better manage host health.

## 1. Microbial niche construction

Microbes engage in a remarkable array of behaviours that shape and change their shared environments (table 1). In short, microbes are prodigious ‘niche constructors' [16–20], with construction varying from elaborate and adaptive extracellular mechanisms that yield collective improvements to a set of focal ‘engineers' (e.g. secreting molecules to enhance nutrient supply and shelter) [21–23], to the inevitable environmental degradation [24–30] (from focal agent's perspective) that follows from the uptake and consumption of limiting resources [18,19,31]. Examples in important pathogens include: the production of the toxin pyocyanin by the opportunistic pathogen *Pseudomonas aeruginosa*, which both kills competitors and is toxic to humans [32,33]; biofilm formation in the nosocomial pathogen *Klebsiella pneumonia*, which greatly enhances its antibiotic resistance [34] and transmission of Shiga toxin encoding phage viruses to commensal microbes by shigatoxinagenic *Escherichia coli*, which can both clear commensal *E. coli* and amplify production of the often deadly Shiga toxin [35,36]. Understanding both the consequences of these behaviours on the host environment and how these consequences shape the ecology and evolution of pathogens is critical in developing robust treatment strategies for these infections.

**Table 1.** Examples of microbial niche construction.

trait	niche construction effect	references
respiration	reduced partial pressure of O_2_-favouring anaerobes	
biofilm production	modifies spatial structure and chemical environment	[1,2]
extracellular enzymes and scavenging molecules (e.g. invertase, proteases, siderophores)	modifies the nutrient environment	[3,4]
resistance to phages	‘herd immunity’ can reduce the effects of phages on others	[5]
antibiotic production	modifies composition of the microbiota	[6,7]
antibiotic detoxification (e.g. β-lactamase)	removes toxic chemicals from environment, alters microbiota	[8]
excretion of metabolic by-products	inhibits or promotes growth of other microbes	[9–11]
immune system suppression	suppression of the immune system may allow growth of other microbes	[12]
immune system activation	provocation of the immune system may clear commensals	[13–15]

Together, the diverse influences of organisms on their environment have been termed ‘niche construction’, an extremely broad term that can be applied to any organismal trait that has some (direct or indirect) consequence for the organism's environment [37,38]. The breadth of this concept has led some authors to question its utility [39–41]. Here, we use a combination of concepts from niche construction and social evolution theory to outline a broad classification of how microbes change their environments, to better understand the ecological, evolutionary and human health implications of these diverse changes.

‘Sociality’ and ‘niche construction’ are strongly overlapping concepts. By broad definitions, they are entirely overlapping ‘theories of everything’ that can describe all biological traits, in so far as all traits affect a focal organism, and other organisms via the inevitable impacts of an organism on its environment. The classification of social behaviours most commonly follows Hamilton [42,43], who classified traits based on lifetime direct fitness consequences of the trait to the actor (the individual expressing the focal trait) and on fitness consequences to recipients (other individuals impacted by the focal trait; table 2; [42,43]). This classification has been extremely influential in evolutionary biology and is increasingly applied to understand adaptation in pathogenic microbes [44–46]. However, the powerful concision of this approach tends to mask the environmental intermediaries that are central to bacterial social interactions (adaptive and non-adaptive) and impacts to the host [47], such as secreted proteins [3,4], metabolites [9–11,48], shared mobile genetic elements [49], changes in pH [23], and changes in host immunity or gut flora [50].

**Table 2.** A Hamiltonian classification of social traits. Traits are classified based on the signs of their lifetime effects on the actor and on recipients, yielding a four-way classification for the trait (behaviour or phenotype) of interest. Note that ‘mutual benefit’ (+/+) here refers to costs and benefits of the trait, and does not necessarily imply ecological mutualism among species. After refs [42,43].

	recipient +	recipient −
actor +	mutual benefit	selfish
actor −	altruistic	spiteful

## 2. Adaptive, maladaptive and incidental niche construction

A common criticism of niche construction theory is that it considers all effects of an organism on its environment as niche construction effects, whereas these effects may not be significant for the evolution of the organismal traits contributing to environmental change [40,41]. As such, the niche construction perspective can be accused of obfuscating the ultimate causes of the evolution of a trait [40,41]. Here, we illustrate that by combining social evolution and niche construction perspectives into a single classification system this limitation can be easily overcome.

Table 3 illustrates this classification of traits depending on their effects on an actor, recipient, and also whether the trait was selected for based on its effects on recipients via modification of the environment. Note that a single actor trait can have distinct classifications depending on the identity of the recipient. This classification differs from the classical Hamiltonian classification of behaviours in that it considers effects on the environment (and therefore other organisms) that were not selected for, i.e. incidental effects. This leads to three broad categories of niche-construction—incidental, maladaptive and adaptive.

**Table 3.** A Hamiltonian classification of niche-constructing behaviours.

	recipient +		recipient −	
	selected for effect on environment	not selected for effect on environment	selected for effect on environment	not selected for effect on environment
actor +	mutually beneficial extended phenotype, e.g. secretions in medium to low densities (EP)	incidental mutually beneficial niche construction, e.g. cross-feeding (INC)	selfish extended phenotype, e.g. intracellular pathogen secretions (EP)	incidental selfish niche construction, e.g. respiration (INC)
actor −	altruistic extended phenotype, e.g. secretions in high density environments (EP)	maladaptive altruism, e.g. inappropriate VF expression (facilitating pathogen invasion) (MNC)	spiteful extended phenotype, e.g. bacteriocin secretions (EP)	maladaptive spite, e.g. inappropriate virulence factor expression (damaging host) (MNC)

### (a) Incidental niche construction

Incidental niche construction (INC) concerns the consequences of ‘self-interested’ traits, traits that increase the direct fitness of a focal individual, and may either increase (*incidental mutually beneficial niche construction*) or decrease (*incidental selfish niche construction*) the fitness of interactants via their effects on the environment that have not been selected for. A large proportion of bacterial interactions fall into the category of *incidental selfish niche construction*, as most metabolic activity will likely degrade the environment for other conspecifics, though metabolic traits are unlikely to have generally been selected for this purpose. However, *incidental mutually beneficial niche construction* is also common (note that this mutualism need not be ecological mutualism). By-products of selfish microbial metabolism may be metabolized by heterospecific microbes, in a process termed cross-feeding or syntrophy [9–11], allowing both interactants to benefit from the selfish metabolic trait even in the absence of any return of beneficial services from the recipient to the actor.

### (b) Maladaptive niche construction

Maladaptive niche construction (MNC) concerns traits that reduce the fitness of the focal individual, and have not been selected based on their effects on the social environment. Such traits would be expected to appear only transiently as they are entirely deleterious to the actor. However, for generalist microbes that are able to survive and grow across a broad range of environments, the potential for locally maladaptive gene expression in novel or rarely encountered environments is increased. A recent comparative analysis of bacterial pathogens of humans illustrated that more generalist, zoonotic species carry a larger genomic repertoire of secreted proteins [4]. This result suggests that generalist bacterial pathogens have larger repertoires of niche-constructing behaviours, and by virtue of their environmental generalism are also more likely to make regulatory ‘bad decisions' in any specific environment—such as within a human host [51]. Inappropriate expression of a virulence factor (VF) in a host species or disease site to which a pathogen is not adapted could lead to negative effects for the focal individual and its neighbours, appearing as *maladaptive spite.* Similarly, production of public goods molecules when they are not of use to the focal individual and relatives, but could be of use to other species, could create the appearance of *maladaptive altruism*.

### (c) Adaptive niche construction or ‘extended phenotypes'

**Figure 1.**
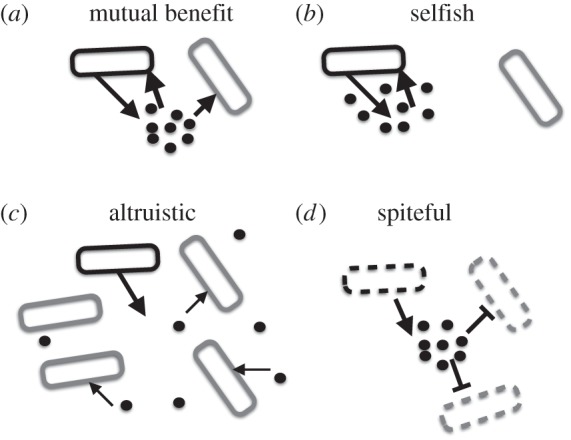
Social calculus for costly secreted factors is dependent on density and mixing of a population. Focal cell and focal cell niche-constructing behaviours are shown in black, interactants are shown in grey. (*a–c*) Secreted public good molecule (e.g. iron-scavenging siderophore or extracellular digestive enzyme). (*d*) Lytically produced anti-competitor toxin (e.g. bacteriocin).

Adaptive niche construction or ‘extended phenotypes' (EP) concerns traits that have been selected for based on their effects on the environment [52]. These traits would be expected to show a clear signature of being designed for their effects on their environment of adaptation (which may not be the human host). For example, for microbes, secreted proteins would be expected to constitute adaptive niche construction traits in their appropriate environment as they are costly to produce and, given that they are secreted, must therefore have been selected for their effects on their environment of adaptation. Adaptive niche construction traits could potentially fall into any of the Hamiltonian classifications of social behaviour (figure 1):

— *Mutually beneficial extended phenotype*, e.g. production of scavenging molecules (siderophores, etc.) at low densities (figure 1*a*). Here, a focal producer receives a net gain owing to the return of iron-loaded siderophores to their producer, as well as gains to other neighbouring individuals [53].
— *Altruistic extended phenotype*, e.g. production of scavenging molecules at high densities in well-mixed populations (figure 1*c*). Here, a focal producer is highly unlikely to receive a direct return on the molecules it has individually produced, but the trait has been selected based on the benefits received by relatives [53].
— *Spiteful extended phenotype*, e.g. suicidal production of bacteriocins (figure 1*d*). Here, a focal individual pays a cost of the trait, but gains an indirect benefit by harming individuals that are less related to them than average [6,7].
— *Selfish extended phenotype,* e.g. production of scavenging molecules when alone or at low densities (figure 1*a*). Here, a focal individual gains from modifying their environment and harms others via environmental degradation (draw-down of shared local resources and/or direct damage to the host) [54].

Adaptive niche construction traits will also inevitably have additional incidental or maladaptive consequences, owing to their impacts on other recipients. For example, adaptive investment in biofilm matrix production can change the physical structure of an environment, producing dramatic changes in flow rates [55], oxygenation [1,2] and can ultimately shift community structure [56–58] and lead to the local extinction of the lineages that initially constructed the biofilm [2].

The expanded classification of social behaviours in table 3, encompassing both social evolution and niche construction perspectives, provides a more holistic view of both the evolutionary causes, and ecological and environmental consequences, of microbial social behaviours. However, a major challenge exists in elucidating to which of these classes any individual microbial behaviour belongs, and how classifications change with changing environmental contexts—for instance, density, mixing, host and microbiome composition (figure 1). However, we believe there are some general guiding principles that can be applied. Whenever an instance of microbial niche construction is caused by secretions/excretions released by microbial cells, the mechanism of production of these secretions can give insights into whether or not this niche construction effect is adaptive in at least some environments. If these secretions are proteins, then we argue that the protein's anticipated niche construction effect must be adaptive in an appropriate environment—why produce a costly large molecule and release it from the cell if its expected net effect on the environment is not beneficial to the actor and kin? This view is also supported by the observation that secreted proteins show a signature of selection for reduced biosynthetic cost [49]. Similarly, if a secreted small molecule is produced by dedicated synthase genes (e.g. siderophores, certain quorum-sensing signal molecules [59]), it stands to reason that it must have some adaptive niche construction function; otherwise, the trait would be lost by selection. However, for excreted molecules that are by-products of selfish intracellular metabolic processes, we argue that the niche-constructing impacts (such as cross-feeding or syntrophic community structures) are likely to be purely incidental effects.

## 3. Utility of combining niche construction and social evolution perspectives: managing bacterial niche construction

The combination of niche construction and social evolution perspectives promises a richer understanding of microbial social behaviours. However, what value does this combination of perspectives offer for our understanding, and ultimately management, of our interaction with microbes?

Consider for example a bacterial VF. VFs are clearly niche-constructing traits, as they are typically defined and identified by their causal impact on host-level disease, in addition to the requirement of being expendable in rich culture. In short, they are molecular determinants of pathology, coded by non-essential genes [60]. What would a niche construction perspective tell us about VFs? The focus of a niche construction perspective would be on the effects of the virulence trait on the environment through space and time. Key questions would be

(1) How is the microbiota affected?
(2) How is the immune system affected?
(3) How spatially diffuse are the effects (VF diffusion, propagation, etc.)?
(4) How temporally elongated are the effects (VF durability, immune memory, etc.)?
(5) How do impacts on immunity and other microbes interact?

On the other hand, a social evolution perspective asks a very different set of questions:

(1) How does VF production affect the producer's fitness?
(2) How does VF production affect the fitness of other individuals?
(3) What is the relatedness between affected individuals and producers?
(4) Ultimately, what was the VF selected for?

The niche construction perspective focuses on the range of effects of the trait on the host environment through space and time, and identifies potential consequences directly for host health and those mediated by microbiota, immune system, etc. This focus on mechanism is more aligned with mainstream microbiological approaches, and allows us to make inference by homology—similar molecules in another organism will likely have similar effects. In sum, the niche construction perspective identifies the full range of *potential* direct and indirect impacts of the trait on the host and microbiota. In contrast, the social evolution perspective tells us which of these effects are important for the evolution of the trait, if any (recognizing the potential for the trait to be sculpted by selection in distinct environments [51,61]). By providing a diagnosis of the selectively relevant forces acting on the VF, a social evolution perspective provides vital information on the potential evolutionary consequences of interfering with the trait in different ways.

### (a) Case study 1: *Clostridium difficile* dynamics in the gut microbiome

As a concrete example, let us consider the recently discovered suppression of *Clostridium difficile* infection by its cogener *C. scindens* [62]. *C. difficile* is a ubiquitous soil-dwelling microbe, and is found in a carriage state in the colon of approximately 2–5% of the adult human population [63]. *C. difficile* is also a major opportunistic pathogen of humans with disrupted gut microbiomes, notably following antibiotic treatment. Antibiotic-associated reductions in the resident commensal microbiota can lead to *C. difficile* colitis [64], a dangerous condition and increasingly the target of controversial ‘faecal transplant’ therapies, where faecal material from healthy donors is used to repopulate the microbiome of the patient [65]—a clear and dramatic example of medical niche construction. While faecal transplants are reported as an empirical success, the search for a mechanistic basis of their efficacy is pressing, in order to replace faecal transplants with defined therapeutic consortia.

Using a combination of metagenomics of the microbiome of hospitalized patients and mouse model infections, Buffie *et al.* [62] recently showed that the presence of *C. scindens* protects against infection with *C. difficile* via the biosynthesis of secondary bile acids, which inhibit *C. difficile* growth. This suggests that the use of *C. scindens* as a probiotic for patients undergoing antibiotic treatment may help prevent debilitating *C. difficile* infections, without recourse to entire faecal transplantation.

**Figure 2.**
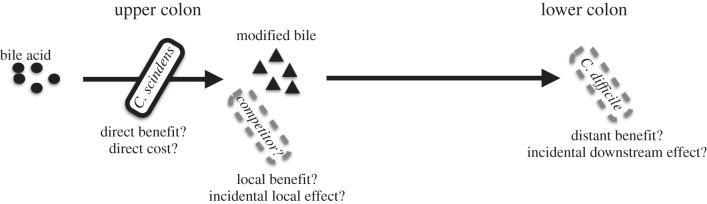
The niche construction effect of bile acid modification by *C. scindens*. Dehydroxylation of bile acids by *C. scindens* at the caecum (upper colon) leads to suppression of *C. difficile* in the lower colon. Bile acid modification may directly yield energy for *C. scindens*, but may also suppress the growth of local competitors, which could be an adaptive or incidental function. Finally, the downstream effect of *C. scindens* on *C. difficile* may either provide benefits via immune system interaction or may be incidental.

What does a joint niche construction and social evolution perspective offer in terms of understanding this potential new treatment? While *C. difficile* infections primarily occur in the lower parts of the colon [66], *C. scindens* and other secondary bile-acid-producing bacteria are thought to be primarily active in the upper parts of the colon [67,68]. Thus, the production of secondary bile acids by *C. scindens* is analogous to the canonical example of niche construction—a beaver dam—in having downstream effects modifying the environment (figure 2).

Whereas the biomedically significant effects of enzymatically modifying bile acid are downstream of the actor, the nature and localization of the costs and benefits to the actor are currently unknown (figure 2). We anticipate that the suppression of *C. difficile* has negligible impacts on *C. scindens* owing to their significant spatial segregation (although immune-mediated interactions are possible). It is more likely that bile acid modification provides local benefits to *C. scindens,* through the chemical suppression of local (and currently unidentified) competitors. Finally, it is possible that dehydroxylation of primary bile acids directly yields energy for *C. scindens*, and all extracellular impacts are incidental for the focal actor. Critical to elucidating which of these scenarios holds will be characterizing the temporal and spatial extent of the costs and benefits of secondary bile acid synthesis to *C. scindens,* its local interactants, and downstream players including *C. difficile.* If bile acid modification is driven by direct metabolic gains (i.e. INC), then the microbiome management goal will be to increase profitability of this reaction (potentially via diet, prebiotics). In contrast, if the trait is driven by local competitive gains, then management might involve manipulating competitive cues driving expression of the enzymatic modification. In either case, understanding the social and non-social selection pressures governing bile acid modification is critical for ensuring any such intervention is robust to potential within host evolution of *C. scindens* and its interactants.

### (b) Case study 2: *Pseudomonas aeruginosa* dynamics in the cystic fibrosis lung microbiome

Cystic fibrosis (CF) is a genetic condition that primarily affects the lung, leading to impaired airway clearance and the establishment of chronic lung infections. Chief among the multiple microbial pathogens of the CF lung is the environmental generalist bacterium and opportunistic pathogen, *Pseudomonas aeruginosa.* In line with other generalist opportunistic pathogens, *P. aeruginosa* is characterized by a broad arsenal of secreted ‘VFs', with the typical genome carrying 60 or more genes coding for secreted proteins, spanning toxins, exoenzymes and immunomodulatory factors [4]. On initial colonization of mammalian tissues, many of these secreted factors are upregulated [69,70]. However, during subsequent evolution within chronically infected CF patients, many of these secreted factors are subsequently lost [71–74]. The loss of collectively produced VFs has led social evolution theorists to suggest that the within-host evolutionary dynamics is due to social interactions favouring non-producing ‘cheater’ strains that exploit the benefits, but do not pay the costs of collective action [75,76]. However, it is also possible that the benefits of VF expression change in time owing to a successional process of environmental modification—in the early stages of lung colonization, secreted factors are beneficial in remodelling the lung environment, and then only later on do they become redundant [51]. Finally, it is possible that these VFs are always redundant in the lung and that their initial upregulation was a regulatory mistake, a ‘bad decision’ resulting from a severe environmental mismatch [51]. The potentially widespread existence of regulatory mistakes in generalist microbes was recently highlighted in a study of *P. aeruginosa* acute burn and chronic wound infections, which found no correlation between gene expression level and selection on a mutant—*P. aeruginosa* is just as likely to upregulate redundant genes and downregulate useful genes as the converse [77].

Understanding the relative roles of these three non-exclusive hypotheses (cheating, succession and redundancy) is important from a management perspective. One emerging therapeutic option to manage *P. aeruginosa* virulence in CF lung infections is the use of ‘anti-virulence’ drugs. Anti-virulence (AV) drugs act by chemically suppressing the expression or functioning of bacterial VFs [60]. If VFs are purely redundant within the lung, then chemically suppressing their activity could even lead to selection *against* resistance [60]. Similarly, if VFs are collectively beneficial and prone to cheater exploitation, then drug treatment could select *against* resistant cells—but only if resistant ‘cooperators' and sensitive ‘cheats' are sufficiently well mixed [60,78]. Finally, if the benefits of VF expression are temporally variable, then the picture becomes yet more nuanced and dependent on the successional stage of the infection process.

### (c) Case study 3: Provocation of the host immune response by *Salmonella enterica* serovar Typhimurium

One of the most dramatic ways in which bacteria can modify the within-host environment they experience is by provoking the host immune system in order to clear competitors [13]. If a pathogen is suitably protected, eliciting a strong immune response could facilitate its invasion by clearing commensals and other competing pathogens from the infection site [14,36]. Many examples of this behaviour have been characterized in bacteria, including provocation of immune response by inflammation-promoting toxins or immunogenic factors in *E. coli* O157:H7 [79,80], *Streptococcus pneumoniae* [81,82] and *C. difficile* [83–85], leading to clearance of commensal competitors, and by recruitment of neutrophils into the paranasal spaces by *Haemophilus influenzae,* leading to the clearance of its competitor *S. pneumoniae* [15]. Interestingly, in this last example, provocation of the immune system by *H. influenzae* may have selected for more virulent serovars of *S. pneumoniae*, which can resist this immune response [86]. While for many of these examples, it is unclear whether this inflammation is adaptive, one of the most well-described examples of adaptive immune provocation occurs in the enteric pathogen *Salmonella enterica* serovar Typhimurium.

Pathogenic salmonella, like other enteric food-borne pathogens, face a dramatic ecological challenge on entering the digestive tract: the presence of a diverse and saturated ecosystem of resident and presumably locally adapted microbes. Stecher *et al*. [13] demonstrated that *S. typhimurium* can overcome resident microbiome competitive suppression by provoking an inflammatory host immune response to preferentially clear commensal competitors. In order to overcome competition from the commensal microbiota, *S. typhimurium* uses type III secretion system 1 (T3SS-1) to manipulate and invade gut tissue and induce inflammation in the host, to which *S. typhimurium* is resistant [13]. Expression of these VFs is regulated by a stochastic bistable switch, with only a subpopulation of *S. typhimurium* expressing the trait. This means that, despite most of the cells invading the gut tissue being killed, cooperators can gain indirect benefits by enhancing the growth of relatives in the gut lumen [87]. However, this behaviour has been shown to also benefit avirulent cheater strains (unable to switch to the virulent, sacrificial phenotype), allowing the cheater strain to increase in frequency within the host [88]. This would initially suggest that provocation of the host immune response is an *altruistic extended phenotype*, implying that anti-virulence drugs [60,89], or even treatment with cheater strains (‘cheat therapy’ [90]), may be an evolutionarily robust way to manage *S. typhimurium* virulence.

However, the picture becomes more complicated when we consider the effects of antibiotic treatment on the evolutionary dynamics of this trait. Ciprofloxacin treatment has been shown to reduce the ability of cheats to invade WT *S. typhimurium* during infection [91,92]. This occurs as *S. typhimurium* cells that have invaded the gut tissue are protected from the effects of the antibiotic, acting as a ‘persister’ subpopulation [91], whereas cheaters and cooperators in the gut lumen are cleared by the antibiotic [91]. A small proportion of the cooperators that have invaded the gut tissue then re-seed the infection in the lumen [91]. These results highlight that under antibiotic treatment the benefits of expression of the virulent phenotype (survival in a persistence state/location) become direct, whereas the environmental modifications likely become redundant (competitor clearance is now driven by antibiotics). In this scenario, the virulent phenotype becomes a case of *incidental selfish niche construction*, and there will be strong selection for resistance to any anti-virulence drug (and within-host selection against cheats), reducing the evolutionary robustness of this treatment strategy. Under antibiotic treatment, a more promising approach may be to try to manage the niche construction effects (i.e. inflammation) caused by tissue invasion.

## 4. Conclusion

The evolutionary management of infectious diseases needs clear focus on both the effects of microbes on their environment and the consequent selection pressures of these effects. Whether adaptive or not, elucidating the niche construction effects of microbes is critical as they are likely to impact on host health. However, we also need to clearly delineate the subset of impacts that have a selective relevance—the direct and indirect costs and benefits of a pathogen trait—as it is this social evolution cost–benefit analysis that governs the trajectories of biomedically significant traits (e.g. drug resistance, virulence, emergence, microbiome resilience) through very rapid evolutionary time.
